# Generation of Highly Homogeneous Strains of Zebrafish Through Full Sib-Pair Mating

**DOI:** 10.1534/g3.111.000851

**Published:** 2011-10-01

**Authors:** Minori Shinya, Noriyoshi Sakai

**Affiliations:** Genetic Strains Research Center, National Institute of Genetics, and The Graduate University for Advanced Studies (SOKENDAI), Mishima, Shizuoka 411-8540, Japan

**Keywords:** *Danio rerio*, sib-pair mating, inbreeding depression, loss of heterogeneity

## Abstract

Genetically homogeneous populations, such as inbred strains, are powerful experimental tools that are ideally suited for studying immunology, cancer, and genetics of complex traits. The zebrafish, *Danio rerio*, has been underutilized in these research areas because homogeneous strains of experimental fish have not been available in tractable condition. Here, we attempted to inbreed two zebrafish wild-type strains, Tübingen and India, through full sib-pair mating. Although the inbred Tübingen strain failed to thrive and was lost after 13 generations, an inbred India strain (IM) has been maintained successfully. The IM strain has endured 16 generations of inbreeding and has maintained a healthy condition. Two additional strains, IM12m and IM14m, were established as closed colonies from the branches of the IM strain. Genotype analyses using genetic markers revealed a dramatic decrease in polymorphisms (62% dropped to 5%) in both IM (generation 14) and the two closed colonies. This indicates a high level of homogeneity in these strains. Furthermore, scale transplantations between individuals within each strain were successful. These data suggest that extremely homogeneous zebrafish strains have been established, thereby creating a valuable resource for practical application.

Genetically uniform strains of animals are valuable tools in biomedical research. Multiple laboratories can use these strains without concern that genetic background variability will confound the experimental results. Indeed, genetic background often affects phenotypes associated with a particular mutant or experimental manipulation in several organisms (Anzai *et al.* 2010; Nojima *et al.* 2004; Nomura 1991; Yamamura *et al.* 2001). In such cases, genetically isogenic strains that can be maintained for generations play an important role in providing highly reproducible results (even several or more years later), and enable us to directly compare and evaluate results obtained from multiple laboratories. Genetically homogeneous strains also provide essential tools for the identification of quantitative loci that affect evolutionarily and biomedically important traits (Frankel 1995; Kimura *et al.* 2007; Klingenberg *et al.* 2004; Klingenberg *et al.* 2001; Tomida *et al.* 2009; Xiao *et al.* 2010). Extremely reliable and reproducible data obtained from genetically homogeneous model organisms enable the dissection of both gene-gene and gene-environmental interaction patterns. Furthermore, to identify the susceptible genes, it is often necessary to generate congenic strains; strains that differ from their inbred partner strain by only a short chromosomal segment. The stably maintained homogeneous strain makes it possible to backcross continuously to generate congenic strains (Markel *et al.* 1997; Morel *et al.* 1996; Yui *et al.* 1996).

Numerous attributes in the zebrafish, *Danio rerio*, including large numbers of eggs per clutch, a short generation time, and relatively low costs associated with maintenance, enable the application of a wide variety of biological techniques to the organism, such as embryonic manipulations, forward and reverse genetics, and molecular biology. Consequently, over the past two decades, the zebrafish has become an important vertebrate model organism in developmental biology, neuroscience, and cancer research. However, the zebrafish system has one major weakness; namely, the lack of highly homogeneous strains that can be stably maintained for many generations. For example, in both mice and medaka, over 10 inbred strains have been maintained by full sib-pair mating for more than 20 generations; these strains have expanded the use of these animals as vertebrate model systems (Naruse *et al.* 2004; Peters *et al.* 2007). Comparable zebrafish inbred strains have not been available to the scientific community.

Streisinger *et al.* (1981) demonstrated that gynogenesis could be used to produce homozygous diploid fish in which only the maternal genome is represented in the offspring. Two zebrafish strains, C32 and SJD, were generated in this manner and were subsequently inbred for a number of generations (Johnson *et al.* 1995; Nechiporuk *et al.* 1999; Streisinger *et al.* 1981). Genetic analysis of single nucleotide polymorphisms (SNPs) revealed that 7% and 11% of tested loci were polymorphic in the SJD and C32 strains, respectively (Guryev *et al.* 2006). As 14.1% of these SNPs were polymorphic in an outbred strain, WIK, it is difficult to conclude that SJD and C32 truly represent homogeneous lines (Guryev *et al.* 2006).

Recently, several additional clonal zebrafish strains have been generated by gynogenesis. Successful transplantations between adult fish have been performed with these strains without severe immune reactions (Mizgirev and Revskoy 2010). It is too early to know, however, whether these strains can be maintained and expanded over many generations. Finally, there is an additional inbred strain, SJA, which is listed in the Zebrafish Model Organism Database (ZFIN, Bradford *et al*. 2011). It is unclear how SJA was created, however, and only 85% of its genome is guaranteed to be monomorphic.

It is clear that some logistical difficulties (*i.e.*, inbreeding depressions), including high mortality levels of embryos and larvae, biased sex ratio, and few eggs from natural crosses, have made it hard to inbreed zebrafish. However, it is not clear whether these difficulties are too severe to establish and maintain homogeneous strains in zebrafish. In this study, we have inbred two zebrafish wild-type strains, attempting to know the severity of inbreeding depression in zebrafish and to establish highly homogeneous zebrafish strains through continuous full sib-pair mating.

## Materials and Methods

### Strains and fish maintenance

Two outbred wild-type strains, India and Tübingen, were used to generate genetically homogeneous strains of zebrafish. The India strain is a strain obtained from expedition to Darjeeling (see ZFIN at http://zfin.org/action/genotype/genotype-detail?zdbID=ZDB-GENO-980210-28). The Tübingen strain originated from a local pet shop and was maintained for many generations in the laboratory at Tübingen (Haffter *et al.* 1996). This strain has been used by the Sanger Institute for the *Danio rerio* Sequencing Project. The two inbreeding strains were named IM (India-Mishima) and TM (Tübingen-Mishima). For the IM strain, male and female fish of generation *n* were denoted I*_n_*-M# and I*_n_*-F#, respectively (# represents the serial number). An IM pair (consisting of one male and one female) within generation *n* was denoted I*_n_*-#. For example, the IM founder pair (I_0_-1) consisted of one male (I_0_-M1) and one female (I_0_-F1). The same nomenclature was applied to TM fish, with a “T” replacing the “I.” The method for fish breeding is described in supporting information, File S1.

### Generation and selection of pairs

**Figure 1:**
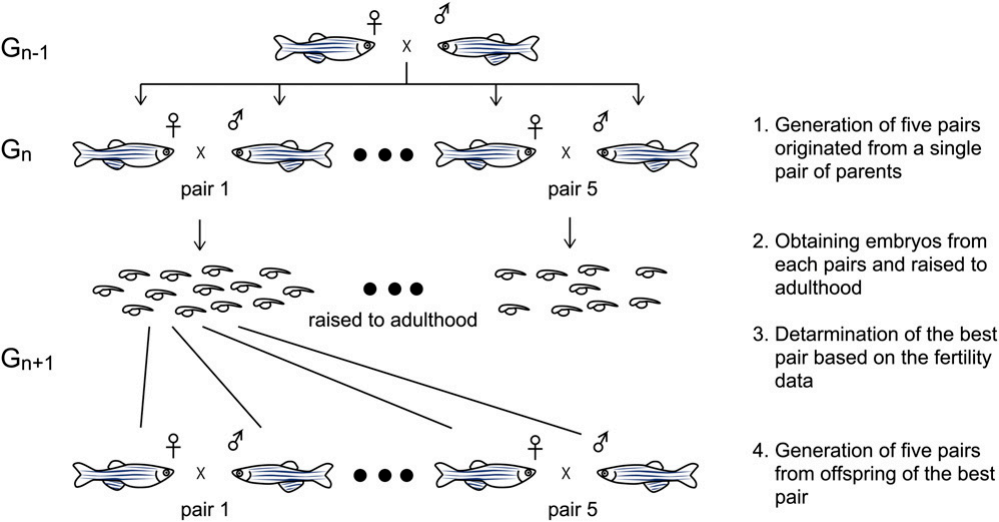
Schematic representation of typical inbreeding strategy. (1) Five pairs at generation n (G*_n_*) were generated from offspring originated from a single pair of fish at generation *n*-1 (G*_n_*_-1_). (2) Embryos (G*_n_*_+1_) from each pairs were obtained and raised to adulthood. Data corresponding to the fertility of each pair were recorded. (3) The best pair at generation *n* (pair 1 in this figure) was determined based on the fertility data (see *Materials and Methods* for detailed criteria). (4) Inbreeding was repeated from (1) for the next generation; *i.e.*, five pairs at generation *n* + 1 (G*_n_*_+1_) were generated from the offspring of the best pair at generation *n*.

To generate genetically homogeneous zebrafish strains, sister-brother mating was performed for many consecutive generations. A basic strategy for inbreeding is schematically represented in Figure 1. Typically, five males and five females were selected from offspring obtained from a single pair of parents. Five single-pair matings were established from them, and embryos from each pair were raised to adulthood. To determine which pair’s offspring was intercrossed to generate the next generation, the following criteria were used: (1) average number of eggs laid; (2) fertilization efficiency; (3) average number of embryos surviving until three days postfertilization (dpf); (4) survival rate of embryos at three dpf; (5) normal development in the most embryos; (6) normal growth into healthy adult fish in the most larvae; and (7) sex ratio. If most pairs of a given generation revealed problems in any of these parameters, additional pairs were generated, and the process was repeated. Paired fish of generation *n* were fixed in 100% ethanol and placed at –30° after confirmation that their offspring were producing viable progeny (generation *n* + 2). If a paired fish was found dead before this confirmation was possible, the deceased fish was fixed in 100% ethanol and immediately placed at –30°. Closed colonies from two distinct branches of the IM family were maintained through mass-mating of healthy-looking females and males (three to eight each). Significant differences between the fertility data at generation *n* and those at generation 0 were tested by *t*-test.

### Genetic monitoring

During the course of the experiment, the fish were genotyped to monitor genetic homogeneity and to guard against contamination. Genotyping was performed using the simple sequence length polymorphisms (SSLP) listed on the ZFIN web site. Positional information for each marker was obtained from ZFIN and the Massachusetts General Hospital Zebrafish server (Shimoda *et al*. 1999). The allele size of each SSLP marker was determined for both TM and IM founder pairs (generation 0), and markers that were polymorphic in the original pair were identified. Furthermore, using four progeny from each strain (generation 1), codominant inheritance of the selected markers was confirmed. Genomic DNA was extracted from fin-clips of living fish or one-quarter of an ethanol-fixed fish using the Maxwell 16 Automated Purification System (Promega, Tokyo, Japan). Each fish’s genotype was determined using methods described in Kimura *et al.* (2005), with some modifications.

### Scale transplantation

Two fish were anesthetized using 0.01% ethyl 4-aminobenzoate (w/v) and placed on a wet Kimwipe with the lateral side up. For autotransplantation, one dorsal and one ventral scale were removed using forceps. The dorsal scale then was inserted into the ventral position (where the ventral scale had just been removed). For transplantations between individuals, one dorsal scale from the donor fish was inserted into the ventral region of the recipient fish. Dorsal scales contain melanophores and, therefore, are easily identified within the recipient’s nonpigmented ventral region. The day after transplantation, the presence of the donor scale was confirmed. If the transplanted scale was missing at this time, the operation was deemed a failure. Between posttransplantation days 3 and 24, the transplanted scale was checked frequently (posttransplantation days 3 to 9, every two days; posttransplantation days 10–24, every three days) to monitor for allograft rejection. Significant differences between autotransplantation and transplantations between individuals were tested by Fisher’s exact test.

## Results

### TM inbreeding

**Figure 2:**
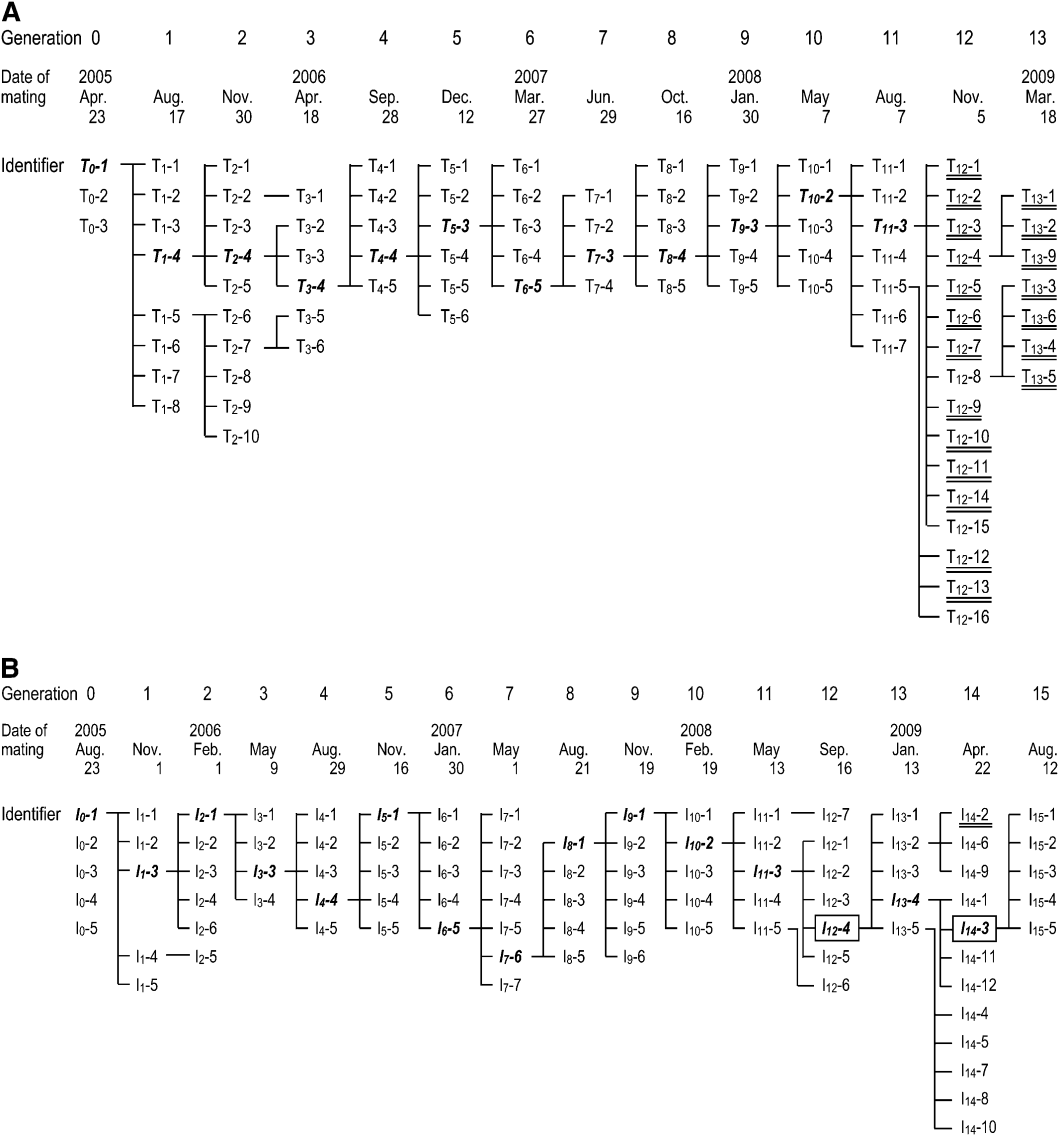
Pedigree of the highly homogeneous strains. (A) Pedigree of the TM strain. (B) Pedigree of the IM strain. Bolded paired identifiers represent the pair whose offspring were kept inbreeding. Single-underlined paired identifiers represent pairs that produced some embryos with the TM phenotype, whereas double-underlined paired identifiers represent pairs that only produced embryos with the TM phenotype. Two closed colonies (IM12m and IM14m) were established from the pairs in boxes.

**Figure 3:**
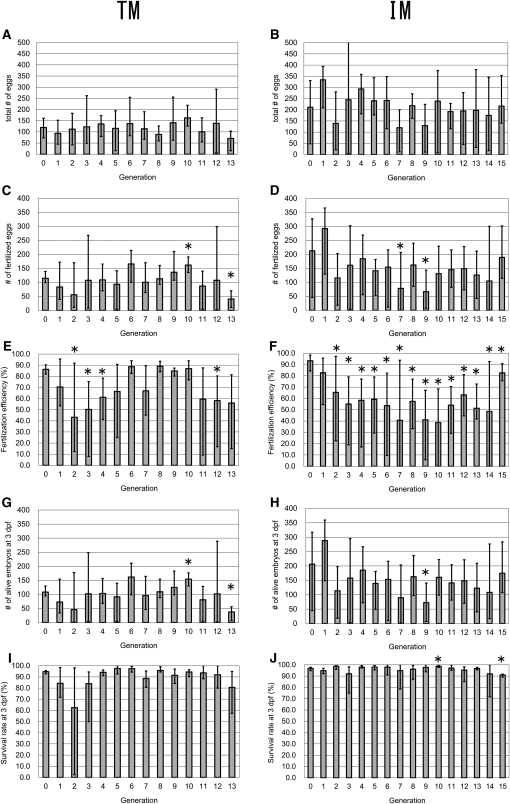
Fertility of the TM and IM strains at each generation. Graphed data from TM (A, C, E, G, and I) and IM (B, D, F, H, and J) are shown. (A, B) Number of eggs per clutch. (C, D) Number of fertilized eggs per clutch. (E, F) Percentage of fertilized eggs per clutch (fertilization efficiency). (G, H) Number of embryos that survived until three dpf per clutch. (I, J) Percentage of embryos that survived until three dpf per clutch. Bars indicate mean values; error bars indicate range of values. Both maximum (up) and minimum (down) values are indicated. Asterisks indicate the significant differences (*P* < 0.05) from the values observed in generation 0.

Tübingen is a heterogeneous, wild-type strain of zebrafish, and in April 2005, inbreeding was initiated with three independent single-pair matings (generation 0) (Figure 2A). Complete fertility records for each pair in each generation are shown in Table S1. The first obstacle to inbreeding arose in generation 2 (Figure 3C, E, G, I). At this point, the average number of fertilized eggs per clutch was 56 (the lowest value measured during the study, except for the value in generation 13), and the fertilization efficiency was only 43.2%, significantly lower than the value at generation 0. At three dpf, the number of living embryos and the survival rate (each measured on a per clutch basis) also were quite low (46 and 62.5%, respectively), although the differences were not significant compared to the data at the beginning of the inbreeding. In this generation, 8 of 10 pairs yielded less than 50 living embryos at three dpf per clutch. Because of generation 2’s fertility problems, additional pair matings were established from generation 1 fish, and two subfamily branches (one originating from T_1_-4 and the other from T_1_-5) were maintained (Figure 2A). Because of these difficulties, it took approximately five months to establish the next generation successfully, whereas the typical zebrafish generation time is three months (Laale 1977) (Figure 2A). After generation 4, no additional major problems arose, and the TM strain was successfully propagated until generation 11 (Figures 2A and 3). The body size and lifespan seemed the same as that of the original strain, although most TM fish were usually disposed of after around one year to save space in the fish facility. However, a biased sex ratio was observed in pair matings of generation 2 (T_2_-2, T_2_-3, and T_2_-8), generation 5 (T_5_-1, T_5_-4), and generation 6 (T_6_-2). In each case, the progeny from the crosses were predominantly male.

**Figure 4:**
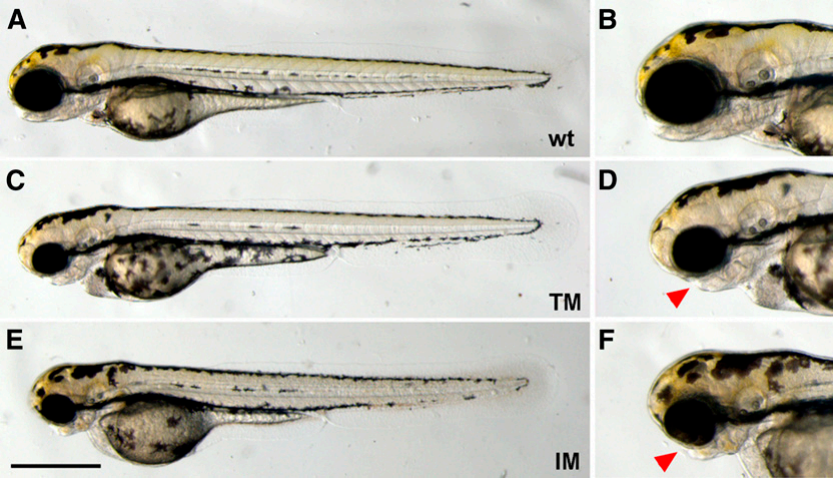
The TM phenotype. Embryos with the TM phenotype had small heads and underdeveloped jaws. Lateral views of the embryos are shown. (A, B) Wild-type embryo. (C, D) TM embryo with the TM phenotype. (E, F) IM embryo with the TM phenotype. Arrowheads mark the underdeveloped jaws. Scale bar is 0.7 mm.

The second major difficulty arose in generation 12 of the TM strain. Most pairs of generation 12 produced embryos with phenotypes that included a small head, an underdeveloped lower jaw, and poor circulation (Figure 4). These phenotypes were collectively designated the “TM phenotype.” Embryos with the TM phenotype died within 10 dpf from cell necrosis within the brain or starvation or both. As a result, additional single-pair matings were established from generation 12 fish. Pairs T_12_-8, T_12_-15, and T_12_-16 produced phenotypically normal embryos, whereas T_12_-4 produced both normal embryos and embryos with the TM phenotype (Figure 2A, Table S1). The remaining 12 single-pair matings, however, only produced embryos with the TM phenotype. Normal embryos obtained from the above single-pair matings were raised to adulthood. Unfortunately, the T_12_-15 and T_12_-16 progeny were all male. In generation 13, therefore, only fish from T_12_-4 and T_12_-8 were available for sib-pair mating (Figure 2A). All progeny from generation 13, however, exhibited the TM phenotype. In a final attempt to overcome this problem, additional single-pair matings were established from generation 11 (T_11_-6 and T_11_-7). Embryos from these crosses appeared normal and healthy; however, all T_11_-7 progeny were male, and the T_11_-6 progeny did not produce any fertilized eggs. Therefore, despite much effort, inbreeding of the TM strain ended at generation 13.

### IM inbreeding

Inbreeding of the IM strain began in August 2005 (Figure 2B). India is a wild-type strain that has been used often as the reference line for mapping mutations (Geisler 2002). Inbreeding of India fish was initiated with five single-pair matings (generation 0). Complete fertility records for each pair in each generation are shown in Table S2. Successful progression from one generation to the next took between two-and-one-half and four months for IM (Figure 2B), indicating a relatively smooth inbreeding process.

As with TM, the fertilization efficiency of the IM strain decreased soon after the inbreeding process was initiated. IM efficiency remained significantly low (approximately 50%, Figure 3F). In contrast, TM fertilization efficiency had recovered by generation 6. However, because IM females tended to lay more eggs than TM females (Figure 3A, B), the number of fertilized eggs and surviving embryos at three dpf were mostly higher in IM compared with TM (Figure 3C, D, G, H). Furthermore, although the value was still significantly lower than that of generation 0, the fertilization efficiency of IM suddenly increased to ∼80% at generation 15, providing hope that this parameter may improve in subsequent generations. The survival rate at three dpf did not vary much during the course of the experiment (Figure 3J). Surprisingly, one pair (I_14_-2) produced embryos with a phenotype indistinguishable from the TM phenotype (Figure 2B, compare Figure 4E, F with Figure 4C, D). Therefore, we established additional single-pair matings using IM fish from some pairs in generation 13 (I_13_-2, I_13_-4, and I_13_-5). To be safe, generation 15 single-pair matings were prepared from I_14_-3, which had different parents than I_14_-2 (Figure 2B). Phenotypically wild-type embryos were obtained from all generation 15 pairs, and the progeny, the IM fish of generation 16, have been growing well. During earlier stages of the IM inbreeding process, there were examples of unbalanced sex ratios. I_3_-1, I_3_-2, and I_7_-1 produced primarily male progeny; however, this did not develop into a persistent problem. In addition, generation 15 and generation 16 fish generally looked a little bit smaller than fish of their original strain, but they appeared healthy in all other ways and consistently produced fertilized eggs without artificial insemination. Just like fish in the India strain, their original strain, most of IM fish were usually in good condition until they were disposed of to surrender space (about one year, normally). We will continue to propagate the IM strain through sib-pair matings.

As was learned from the TM strain, continuous sib-pair mating carried the risk of strain extermination. To avoid complete loss of the genetically homogeneous IM strain, therefore, we also established two IM branch families and maintained them as closed colonies. I_12_-4 and I_14_-3 served as founder pairs of these colonies (Figure 2B), and they were named IM12m and IM14m, respectively. These strains have been successfully propagated for two to four generations by mass mating.

### Genetic assessment of the strains inbred

To monitor inbreeding progress and to avoid strain contamination, the pairs whose progeny was crossed in the next generation were frequently used in a genetic analysis. Each fish was genotyped using two genetic markers per chromosome (50 markers in all). These 50 markers were prepared for the TM and IM strains as listed in Table S3 and Table S4, respectively. Polymorphic markers in founder fish were included in the marker sets as much as possible. In generation 0 of the TM and IM strains, polymorphisms were detected at 52% and 84% of the examined loci, respectively (Table 1). By generation 9, the number of polymorphic makers had decreased to 12% (TM) and 8% (IM), indicating a dramatic loss of genetic diversity. In the TM strain, 8% of the markers were polymorphic at generation 11, whereas in IM, only 2% of the markers were polymorphic by generation 13. No unexpected alleles were detected in this analysis, suggesting a lack of contamination during the inbreeding process.

**Table 1:** Number of polymorphic markers within the TM and IM strains

			Generation					
Marker Set	Strain		0	9	11	13	14	15
TM marker set (n = 50)	TM	n	26	6	4	ND	ND	ND
		(%)	52.0	12.0	8.0	ND	ND	ND
IM marker set (n = 50)	IM	n	42	4	ND	1	1	1
		(%)	84.0	8.0	ND	2	2.0	2.0
IM marker set + additional marker set (n = 100)	IM	n	62	ND	ND	ND	5	ND
		(%)	62.0	ND	ND	ND	5.0	ND

ND, not determined.

To determine more precisely which genomic regions remained polymorphic within the IM strain, an additional 50 genetic markers were analyzed. This “additional marker set” contained two more markers per chromosome than the original IM marker set. The I_14_-3 pair, which consisted of I_14_-M3 and I_14_-F3, was chosen for this analysis. In addition, three fish from IM14m that had been mass-mated for two generations (IM14m2), three fish from IM12m that had been mass-mated for one generation (IM12m1), and three fish from IM12m that had been mass-mated for three generations (IM12m3) were included in the analysis. The 100 markers covered the zebrafish genome with an average interval of 19.4 ± 12.3 cM. The alleles found in these fish are shown in Table 2. At generation 0, 62% of the markers were polymorphic, whereas only 5% were polymorphic in I_14_-3 (Table 1) and IM12m1 (data not shown). Alleles that remained polymorphic at late stages of inbreeding included: Z6802 at LG1; Z191 at LG7; Z6867 at LG8; Z6895 at LG15; and Z4003 at LG23. Four of the five markers (except for Z6895 at LG15) also were polymorphic in IM12m3. In addition, four of the five markers (except for Z6867 at LG8) were polymorphic in IM14m2. For each of these IM strains, therefore, ∼95% of the genome was homozygous. As such, we have successfully generated highly inbred and genetically homogeneous strains of zebrafish by sequential sib-pair mating.

**Table 2:** Alleles present in IM (generation 14), IM14m2, and IM12m3

LG	Marker*^a^*	Position (cM)	Allele	LG	Marker*^a^*	Position (cM)	Allele
1	**Z9394**	8.5	279	14	**Z9857**	10.5	215
	Z5508	24.2	208		Z5435	34.6	110
	Z6802	61.3	187, 191		**Z8801**	55.5	229
	**Z6223**	85.1	210		Z11837	91.9	133
2	**Z27170**	12.9	282	15	**Z6312**	9.7	188
	Z9361	43.1	211		Z6895	49.3	128, 132*^c^*
	**Z10838**	57.0	205		**Z9773**	85.2	212
	Z9037	86.2	119		Z10193	97	151
3	**Z11244**	14.0	230	16	**Z14570**	3.6	184
	Z15457	40.9	128		Z9881	27.7	195
	Z22516	64.9	155		**Z9685**	43.4	192
	**Z7486**	100.6	181		Z9269	78.9	251
4	**Z1366**	10.9	106	17	Z6010	2.3	138
	Z20533	26.0	206		**Z22674**	33.5	162
	Z11250	44.9	147		Z9692	64.5	251
	**Z7524**	61.6	96		**Z21144**	81.1	167
5	**Z15414**	3.6	190	18	Z11685	4.3	223
	Z8921	28.2	255		**Z9231**	26.2	267
	Z9871	54.0	181		Z8343	49.4	197
	**Z9185**	103.0	86		**Z7961**	75.4	182
6	**Z1265**	3.5	104	19	**Z4009**	12.7	242
	Z880	25.2	170		Z4825	28.1	243
	**Z10183**	51.3	240		**Z7686**	48.8	162
	Z9230	81.0	186		Z9512	80.8	153
7	Z191	1.9	120, 122	20	Z6804	7.9	184
	**Z9133**	35.5	132		Z10056	38.9	259
	Z1239	70.6	186		**Z21067**	64.9	188
	**Z13936**	85.8	190		**Z21485**	115.8	146
8	**Z1637**	4.9	101	21	Z58858	1.4	127
	Z11001	39.9	144		**Z6869**	49.4	129
	Z6867	62.3	220, 236*^b^*		Z1497	119	251
	**Z9279**	81.5	151		**Z6537**	125.2	110
9	Z8348	8.3	263	22	Z9516	6.8	177
	**Z11785**	48.8	190		**Z13794**	26.7	225
	Z5564	74.5	236		**Z3286**	59.2	89
	**Z7564**	86.7	151		Z4682	68.7	292
10	Z10251	8.6	221	23	Z1668	0	149
	**Z8146**	27.9	147		**Z4003**	16.6	243, 247
	Z3835	48.7	153		Z5141	36.6	88
	**Z7558**	77.3	150		**Z4120**	60.1	137
11	**Z22552**	0.0	137	24	**Z10961**	1.2	132
	Z13411	22.6	236		Z23011	36	220
	Z13666	51.4	273		Z13229	53.5	159
	**Z11809**	69.5	131		**Z6923**	75.7	100
12	Z7135	6.3	170	25	**Z21302**	4.3	158
	**Z9891**	32.0	186		Z1197	10.9	210
	Z4830	55.0	158		**Z21055**	42.5	133
	**Z1312**	78.7	147		Z1431	67.8	172
13	**Z9878**	4.7	268				
	Z6104	25.2	179				
	Z17223	51.5	125				
	**Z6007**	80.7	128				

LG, linkage group.

a Markers belonging to the IM marker set are in bold.

b The allele in IM14m2 was 220.

c The allele in IM12m3 was 132.

### Tolerance for tissue transplantation within the IM strain

**Figure 5:**
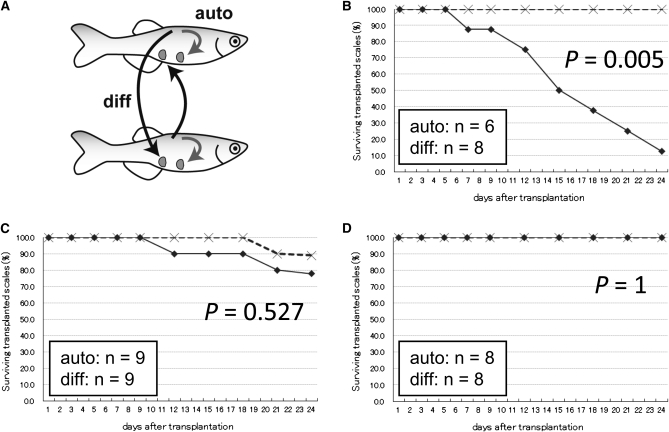
Scale transplantation experiments. (A) Schematic representation of scale transplantation. A dorsal scale was always transplanted to the ventral side. The “diff” (black arrows) indicates transplantation between individuals, and “auto” (gray arrows) indicates transplantation within the same fish. (B–D) The percentage of surviving transplanted scales during the 24 days after transplantation. Crosses indicate autotransplantation, and diamonds indicate transplantation between individuals. Transplantation data from the India outbred strain (B), IM fish at generation 14 (C), and IM fish at generation 16 (D) are shown. *P*-values were calculated from the data at 24 days after transplantation.

As with other animals, adult zebrafish that are part of an outbred colony reject transplanted tissue from another individual (Figure 5B). In mice, however, successful tissue transplantation between individuals occurs if the two mice originated from the same inbred strain (Medina 2010). Similar results have been seen in medaka after 8 generations of sib-pair mating (Hyodo-Taguchi and Egami 1985). To test whether tissue transplantation between adult individuals was possible in zebrafish strains inbred for many generations, scale transplantation experiments were performed (Figure 5A). IM animals from generations 14 and 16, as well as IM12m1, were chosen for analysis. In autograft experiments (in which a dorsal scale is place in a ventral region of the same individual), 86–100% of the autografted scales remained at the transplanted region 24 days after transplantation (Figure 5B–D). In contrast, most grafts between individuals of the outbred India strain were rejected and, therefore, not detected except for one scale at 24 days after transplantation (Figure 5B, *P* = 0.005 by Fisher’s exact test). When scales were transplanted between IM individuals (generation 14), 78% of the scales survived 24 days (Figure 5C); this did not represent a significant difference from autograft experiments (*P* = 0.527 by Fisher’s exact test). For both I_16_ (Figure 5D) and IM12m1 (data not shown), no grafted scales were rejected (*P* = 1 by Fisher’s exact test). These strains of zebrafish inbred for 12 or more generations are, therefore, sufficiently homogeneous to support transplantation of cells between adult individuals.

## Discussion

In this study, we inbred two wild-type zebrafish strains, TM and IM, by full sib-pair mating. Although the TM strain was lost after 13 generations, the IM strain successfully produced offspring from pairs at generation 15; that is, the IM strain endured 16 generations of sib-pair mating. Furthermore, two additional strains (IM12m and IM14m) were established as closed colonies. Genotype analysis revealed that polymorphisms associated with the IM founder pair (62% of the interrogated genetic markers) were dramatically reduced (5%) in IM, IM12m, and IM14m. These data suggest a high level of genetic homogeneity within each strain. In fact, allograft rejection was not observed between individuals within these strains. Together, the IM, IM12m, and IM14m strains represent important resources for the zebrafish scientific community, particularly in the fields of complex trait analysis. Furthermore, the inbreeding strategy described here will be a good reference to establish more strains of zebrafish that are highly homogeneous. Those strains are available from M. Shinya by request.

### Highly homogeneous zebrafish strains: IM, IM12m, and IM14m

The genetic analysis of SSLP markers showed that the genomes of the IM, IM12m, and IM14m strains were ∼95% nonpolymorphic (Tables 1 and 2). The coefficient of inbreeding is the probability that two alleles at a randomly chosen locus in an individual are identical by descents, and it is theoretically calculated as 0.951 for an individual generated by full sib-pair mating for 14 generations (Falconer 1989). Therefore, our estimation that ∼95% of the genomes were homozygous in the IM and its related strains is consistent with the theoretical value, even though we examined only 100 loci. To determine more precisely the degree of polymorphism within these strains, it will be necessary to genotype more genetic markers, including SNPs. Alternatively, direct sequencing of these strains will provide the most accurate assessment of genetic homogeneity. In this regard, we have begun sequencing two genomes from I_14_-3 (I_14_-M3 and I_14_-F3), which will identify polymorphisms within the IM strain.

Highly homogeneous strains of zebrafish are typically difficult to manipulate and maintain. Problems associated with these strains include physiological weakness, biased sex ratios, and the inability to perform natural crosses. Homozygous diploid zebrafish lines C32 and SJD (Johnson *et al.* 1995; Streisinger *et al.* 1981) were shown to be homozygous in over 90% of genomes through SSLP marker analyses (Nechiporuk *et al.* 1999). Unfortunately, C32 lacked vigor, and SJD progeny were predominantly male. Reciprocal introgression of genes to overcome these phenotypes was attempted by crossing C32 and SJD (Rawls *et al.* 2003). The IM strain and two related closed colonies were shown to be ∼95% homozygous. As a result, they are somewhat weaker physiologically than heterogeneous populations (*e.g.*, lower tolerance for sudden changes to pH or temperature). These weaknesses, however, have not hindered their propagation or maintenance. Furthermore, all three strains are capable of laying fertilized eggs under natural-cross conditions. While inbreeding IM, we occasionally observed biased sex ratios. In generations 3 and 7, some pairs predominantly yielded males (see *Results*), but after generation 8, no further gender bias was detected. No sex ratio bias has been seen for either IM12m or IM14m. Taken together, IM strains do not exhibit the phenotypic weaknesses that typically plague homogeneous populations of zebrafish. Careful selection for those phenotypes in each generation may have contributed to this successful outcome.

The genetic homogeneity and health condition of IM, IM12m, and IM14m make them ideally suited for experimental manipulation. Testicular cell grafting experiments have already been performed using these strains (Kawasaki *et al.* 2010). To effectively utilize these strains in genetic analyses of quantitative traits, however, it will be necessary to generate at least one additional inbred strain from a different genetic background (*i.e.*, not India). We have begun additional inbreeding by following the inbreeding strategy described here with some modifications to attempt to generate more homogeneous lines from other zebrafish wild-type strains.

### TM phenotype

TM inbreeding failed at generation 13 because of the TM phenotype (Figure 4). This phenotype arose so suddenly and with such a high frequency (almost every embryo from generation 12 breeding pairs was affected) (Figure 2A) that propagation of the line was not possible. Although TM and IM were raised under essentially identical conditions, the TM phenotype primarily affected only one line. It is likely, therefore, that a genetic factor(s) caused this condition. Phenotypes associated with maternal contributions often arise quickly, but the TM phenotype does not seem to be inherited maternally. One female (T_12_-F13) produced embryos with the TM phenotype when crossed with T_12_-M13 but yielded wild-type embryos when crossed with T_12_-M14 (Table S1). The pattern of phenotypic appearance within the TM family (rapid and essentially ubiquitous) makes it difficult to explain this phenomenon with a single factor. Many factors (perhaps including environmental factors) may have been involved in the phenotypic expression. Genomic comparisons between zebrafish parents that produce embryos with either the wild-type or TM phenotype may lead to identify the causative loci. As we have sampled most pairs for DNA preparation, we are now planning to perform the genomic analyses using those samples to elucidate the genetic basis of the TM phenotype.

The TM phenotype was also seen in a single cross of the IM strain, which was derived from a completely different genetic background. Furthermore, similar phenotypes have been observed in another wild-type strain, AB, which was established long ago in Oregon (H. Yokoi, personal communication). These observations suggest that the TM phenotype is generally associated with increased homozygosity of the zebrafish genome. “Inbreeding depression” is a term used to describe the reduced fitness of a given population that results from breeding related individuals (Charlesworth and Willis 2009). The TM phenotype exemplifies inbreeding depression. Then, in addition to the inbreeding problems described above, how to cope with the TM phenotype is an important issue to establish inbred strains in zebrafish. One way to avoid extinction caused by TM phenotype is the way taken in the IM inbreeding at generation 14: generating many branches and for the next generation, selecting a single-mating pair whose parents are different from those of pairs with TM phenotype (Figure 2B). Alternatively, we can avoid the phenotype, if the causative loci are identified. In this sense, the genomic analyses proposed above will provide a big help to establish inbred strains in zebrafish more easily.

### Level of inbreeding depression in zebrafish

Inbreeding depression presents a major difficulty in generating and maintaining highly homogeneous strains. The degree of inbreeding depression varies from species to species. Quail, for example, exhibit remarkably strong inbreeding depression (Sato 1986). The lethal equivalent (LE) is an index that attempts to quantify inbreeding depression. An LE is defined as a set of alleles that would cumulatively cause an individual to fail to produce its offspring, if all alleles in the set are homozygous in the individual (Morton *et al.* 1956). The number of LEs in quail has been estimated at 8.7 (Sittmann *et al.* 1966), whereas values of 1.4 to 3.6 LEs have been reported for zebrafish (McCune *et al.* 2002; McCune *et al.* 2004). It seems clear, therefore, that zebrafish exhibit less inbreeding depression than birds, a fact that contributed to the success of our IM project.

In both mice and medaka, inbreeding depression is weak enough to allow 20 or more generations of continuous sib-pair mating. However, there is a clear difference between these two species. Inbred strains of medaka are generated quite easily, as only two to three single-pair matings are typically established and tested per generation (Taguchi 1980). In contrast, sibling crosses in mice generate many sterile animals and require numerous single-pair matings at each generation, especially the first generation (Silver 1995). This inbreeding depression typically begins to diminish by generation 8. Therefore, inbreeding depression is stronger in mice than in medaka. In this study, we typically established five single-pair matings per generation. Although we lost one strain, IM remains maintained by continuous sib-pair mating. These data suggest that inbreeding depression in zebrafish is greater than in medaka but likely less than in mice.

Inbreeding depression is caused by increased homozygosity for a single locus with heterozygous advantages and/or a single or multiple deleterious mutations with recessive effects (reviewed in Charlesworth and Willis 2009). It is interesting and important to identify the genomic factors involved in inbreeding depression and in its variation among species. Although some genetic analyses have been performed for the traits related to fitness (Remington and O’Malley 2000), they have not identified those factor(s) yet. Together with the quantitative trait loci (QTL) analyses for the fitness traits, genomic sequencing analyses using TM and IM fish in some generations might give some insights into the factor(s) related to inbreeding depression. Those kinds of data from several organisms will provide information about the genomic factor(s) that contribute to the variation of inbreeding depression among those species.

### Generation of zebrafish inbred strain by full sib-pair mating

In mice and rats, an inbred strain is defined as a population that was established with a single ancestral pair and has been inbred (brother × sister) for 20 or more consecutive generations (Guidelines for Nomenclature of Mouse and Rat Strains). This definition also is used by the medaka community (Taguchi 1990). Although homogeneous lines have been generated in zebrafish through gynogenesis (Johnson *et al.* 1995; Streisinger *et al.* 1981), no zebrafish strains have been established that meet the inbred strain definition used by the mice and medaka scientific communities. To our knowledge, the 16 generations of continuous sib-pair mating that characterize the IM strain represent the longest such effort to date. With 4 more generations of careful inbreeding (for a total of 20 generations), the IM strain will become the first inbred strain of zebrafish that meets the definition of inbred strain used for mice and medaka.

## Supplementary Material

Supporting Information
