# Supplementary material for: Generation of Highly Homogeneous Strains of Zebrafish Through Full Sib-Pair Mating
Source: G3 (Bethesda). 2011 Oct 1;1(5):377–86. doi: 10.1534/g3.111.000851 (PMC3276154; doi:10.1534/g3.111.000851)
Supplement: Supporting Information [file supp_1_5_377__index.html]

Supporting Information 

# Generation of Highly Homogeneous Strains of Zebrafish Through Full Sib-Pair Mating

## Supporting Information for Shinya and Sakai, 2011

**Files in this Data Supplement:**

- Supporting Information - File S1 and Tables S1-S4 (PDF, 48 KB)
- File S1 - Fish  breeding  method  (PDF, 48 KB)
- Table S1 - Fertility record of TM strain (.xls, 88 KB)
- Table S2 - Fertility record of IM strain (.xls, 96 KB)
- Table S3 - TM marker set (.xls, 36 KB)
- Table S4 - IM marker set (.xls, 32 KB)
