# Supplementary material for: Generation of Highly Homogeneous Strains of Zebrafish Through Full Sib-Pair Mating
Source: G3 (Bethesda). 2011 Oct 1;1(5):377–86. doi: 10.1534/g3.111.000851 (PMC3276154; doi:10.1534/g3.111.000851)
Supplement: Supporting Information [file supp_1.5.377_000851.pdf]

File S1

Fish breeding method

Fish were maintained in an in-house facility at 27° in a constant re-circulating system. A light cycle consisting of 14 h of light and 10 h of dark was used. Fish were fed twice each day with dry flake food (Tetra, Melle, Germany) and *Artemia nauplii* obtained by incubating brine shrimp (*Artermia salina*) cysts according to the manufacturer's protocol (Japan Pet Drugs Co., LTD., Tokyo, Japan). System water was prepared by adding sea salt (SEALIFE, MARINETECH Co., LTD., Tokyo, Japan) and sodium bicarbonate to reverse-osmosis water so as to maintain conductivity between 200 and 400 µS (microSiemens) and pH ~7.3. Collected embryos were maintained in autoclaved tap water with methylene blue at 28°. At 4 days post fertilization (dpf), 50–80 larvae were placed in a 150-mm Petri dish containing system water and fed *Tetrahymena thermophila* once per day. *Artemia nauplii* were added to the feed after 6 dpf. When most larvae were eating *Artemia nauplii* (typically 10–16 dpf), the larvae were transferred into tanks with flowing water and fed flake food and *Artemia nauplii* twice per day.

Tables S1-S4

Tables S1-S4 are available for download as Excel files at

http://www.g3journal.org/lookup/suppl/doi:10.1534/g3.111.000851/-/DC1.

**Table S1**	**Fertility record of TM strain**
**Table S2**	**Fertility record of IM strain**
**Table S3**	**TM marker set**
**Table S4**	**IM marker set**
